# Supplementary material for: Iron Release Profile of Silica-Modified Zero-Valent Iron NPs and Their Implication in Cancer Therapy
Source: Int J Mol Sci. 2019 Sep 4;20(18):4336. doi: 10.3390/ijms20184336 (PMC6770483; doi:10.3390/ijms20184336)
Supplement: Supplementary file 1 [file ijms-20-04336-s001.pdf]

1. Materials and Methods

1.1 Cell culture

hNOK (normal human oral keratinocytes) was isolated from health donor and was cultured in Keratinocyte-SFM supplemented EGF, bovine pituitary extract (Gibco) and antibiotics-antimicotics (Gibco) at 37 ℃ under 5% CO_2. The primary cells were obtained with permission from Institutional Review Board of the Cheng-Kung University Hospital (No. B-ER-104-125) and under informed consent of the donors.

1.2. Lysosome membrane permeability assay

The OEC-M1 cells were seeded at a density of 150,000 cells per well in 6-well dishes overnight to allow attachment. The cells were then cultured with ZVI@mSiO2 NPs for 1, 4, and 8 hours, and 5 μM Acridine Orange (AO) was added at the end of the time course, with further incubation for 1 hour [1]. The cells were then harvested and analyzed immediately. The production of AO fluorescence was monitored using FACS Canto II (excitation wavelength, 488 nm; emission wavelength, 515–545 nm), and the results were analyzed using FACS DIVA software and overlay by winMDI v2.9 software.

1.3. Cell death analysis

OEC-M1 cells were cultured in a 6-well plate at a density of 150,000 cells per well. The cells were harvested at 1, 4, and 8 hours after they had been treated with ZVI@mSiO_2 NPs (10 μg/mL). The harvested cells were stained with annexin V and propidium iodide (Annexin V-FITC Apoptosis Detection Kit; BD) for 10 minutes, and apoptosis analysis was then conducted using flow cytometer and software (FACS Canto II; BD).

1.4. Caspase avtivity assay

OEC-M1 cells were cultured in a 96-well plate at a density of 5,000 cells per well. The cells were treated with 1, 5, 10, 50 μg/mL ZVI@mSiO_2 NPs for 12 or 24 hours, and the caspase activity was measured by Caspase-Glo® 3/7 Assay kit (Promega). Briefly the reagents were 1:1 volume ratio added into the tested wells and incubation for 1 hour at room temperature. Then the luminescent were measured by SpectraMax iD5 Multi-Mode Microplate Reader (Molecular Devices).

2. Results

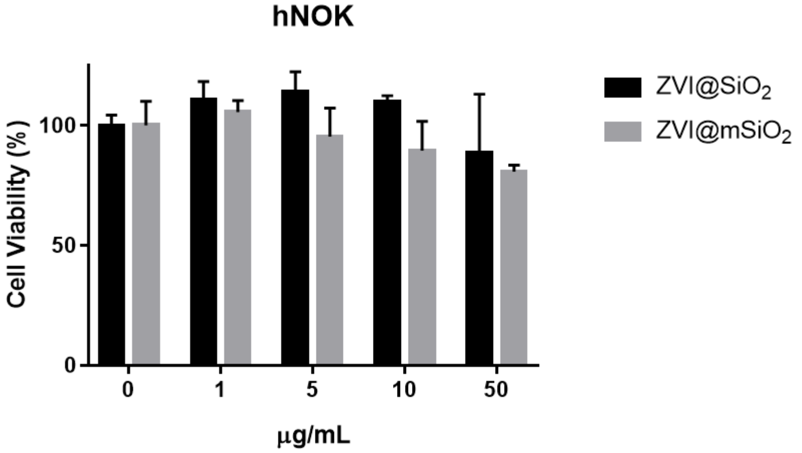

Figure S1. The cytotoxicity of ZVI@SiO_2 and ZVI@mSiO_2 NPs in normal human oral keratinocyte (hNOK) analyzed by MTT assay. Both ZVI@SiO_2 and ZVI@mSiO_2 NPs showed no significant cytotoxicity to hNOK after 24-hours treatment.

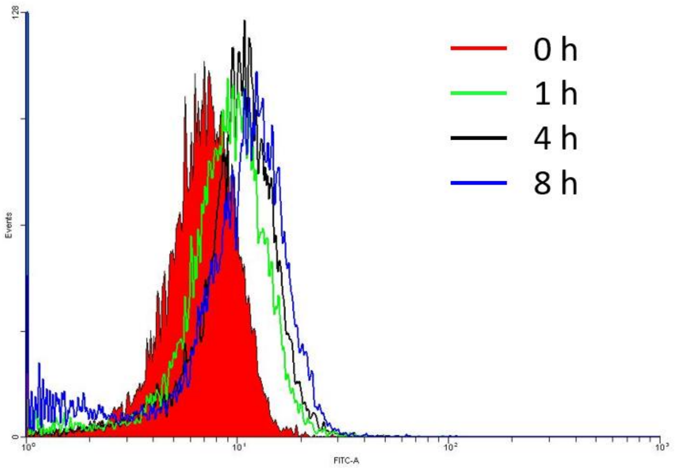

Figure S2. ZVI@mSiO2 NPs significantly increase cancer cell lysosome membrane permeability immediately after 1-hour treatment that may lead to iron ions to escape from lysosome to cytosolic space.

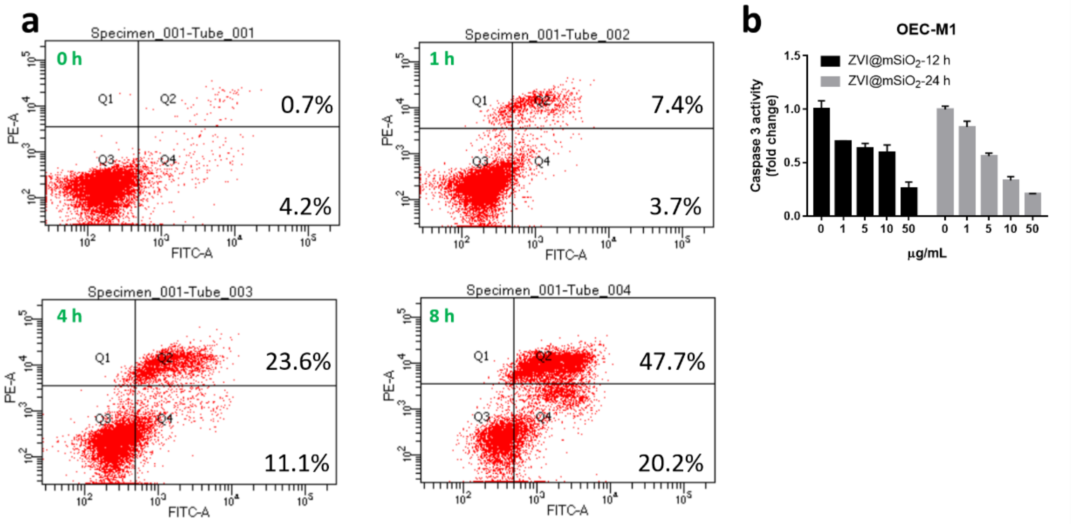

Figure S3. ZVI@mSiO2 NPs induced necrotic cell death in OEC-M1 cells. (a) The double positive populations were dramatically increased as early as the first hour, whereas the minor increase of the Annexin V positive and PI negative population was observed after 4-hour treatment. (b) There was no observable caspase 3 activity of ZVI@mSiO2 treated cells at 12- and 24-hour treatment.

Reference

1. Mijatovic, T.; Mathieu, V.; Gaussin, J.F.; De Neve, N.; Ribaucour, F.; Van Quaquebeke, E.; Dumont, P.; Darro, F.; Kiss, R. Cardenolide-induced lysosomal membrane permeabilization demonstrates therapeutic benefits in experimental human non-small cell lung cancers. *Neoplasia* **2006**, *8*, 402–412. doi:10.1593/neo.05850.
